# Rapid Progress in Immunotherapies for Multiple Myeloma: An Updated Comprehensive Review

**DOI:** 10.3390/cancers13112712

**Published:** 2021-05-31

**Authors:** Hiroko Nishida

**Affiliations:** 1 Department of Pathology, Keio University, School of Medicine, Tokyo 160-8582, Japan; hiroko@a2.keio.jp; Tel.: +81-3-5363-3764; Fax: +81-3-3353-3290; 2 Division of Hematology, Department of Internal of Medicine, Keio University, School of Medicine, Tokyo 160-8582, Japan

**Keywords:** multiple myeloma, belanatmab mafadotin, ADC, BCMA, immunotherapies, bispecific antibody, BiTE, autologous CAR-T cell, allogenic CAR-NK cell

## Abstract

**Simple Summary:**

Despite rapid advances in the development of novel agents over the last decade for the treatment of multiple myeloma (MM), MM remains an incurable disease. Therefore, the development of novel targeting therapies with different mechanisms of action is needed to achieve a deep and durable response for the cure of MM. Recently, an antibody-drug conjugate (ADC), belanatmab mafadotin, which targets B cell membrane antigen (BCMA) on plasma cells, was approved for the treatment of relapsed or refractory MM in 2020. To date, immunotherapies including bi-specific or tri-specific antibodies, adoptive cellular therapy using autologous chimeric antigen (CAR)-T cells, allogeneic CAR-natural killer (NK) cells, and checkpoint inhibitors have been developed for MM, and a variety of clinical trials are currently underway or planned. This review presents an update on the most recent clinical and preclinical advances with a focus on results from clinical trials in progress with BCMA-targeted immunotherapies or the development of other novel targets in MM.

**Abstract:**

Despite rapid advances in treatment approaches of multiple myeloma (MM) over the last two decades via proteasome inhibitors (PIs), immunomodulatory drugs (IMiDs), and monoclonal antibodies (mAbs), their efficacies are limited. MM still remains incurable, and the majority of patients shortly relapse and eventually become refractory to existing therapies due to the genetic heterogeneity and clonal evolution. Therefore, the development of novel therapeutic strategies with different mechanisms of action represents an unmet need to achieve a deep and highly durable response as well as to improve patient outcomes. The antibody-drug conjugate (ADC), belanatmab mafadotin, which targets B cell membrane antigen (BCMA) on plasma cells, was approved for the treatment of MM in 2020. To date, numerous immunotherapies, including bispecific antibodies, such as bispecific T cell engager (BiTE), the duobody adoptive cellular therapy using a dendritic cell (DC) vaccine, autologous chimeric antigen (CAR)-T cells, allogeneic CAR-natural killer (NK) cells, and checkpoint inhibitors have been developed for the treatment of MM, and a variety of clinical trials are currently underway or are expected to be planned. In the future, the efficacy of combination approaches, as well as allogenic CAR-T or NK cell therapy, will be examined, and promising results may alter the treatment paradigm of MM. This is a comprehensive review with an update on the most recent clinical and preclinical advances with a focus on results from clinical trials in progress with BCMA-targeted immunotherapies and the development of other novel targets in MM. Future perspectives will also be discussed.

## 1. Introduction

Multiple myeloma (MM) is a B cell malignancy characterized by an expansion of clonal plasma cells in the bone marrow (BM) with the production of an excess of monoclonal immunoglobulins (M-protein), progressive immune dysfunction, and osteolytic bone disease [1]. It is proceeded by a premalignant disorder known as monoclonal gammopathy of undetermined significance (MGUS), which develops from normal plasma cells via primary genetic events and evolves into smoldering MM, eventually progressing to active MM through secondary genetic events such as genetic abnormalities, epigenetic abnormalities and cytogenetic abnormalities [1,2,3].

**Figure 1:**
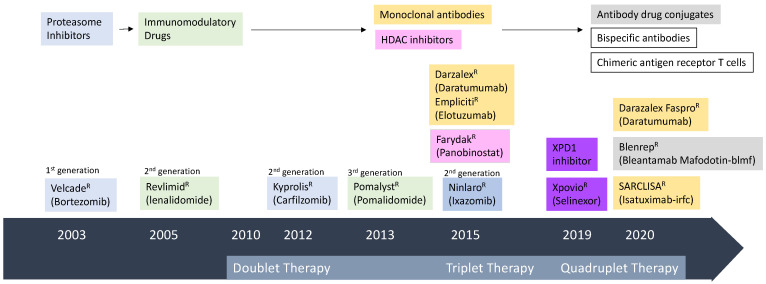
Timeline of world-wide drug development and approval in the treatment of multiple myeloma (MM).

The treatment approaches in MM have exponentially increased over the last two decades with the emergence of the proteasome inhibitors (PIs), immunomodulatory drugs (IMiDs), and monoclonal antibodies (mAb) in addition to autologous hematopoietic stem cell transplantation (auto-HSCT), which have substantially improved both response rates and the survival of MM patients [4,5,6,7,8,9], followed by the development of the second generation of novel PIs; carfilzomib [10,11,12], ixazomib [13,14] and IMiDs; lenalidomide and pomalidomide [15,16,17] (Figure 1). The first-generation IMiD, thalidomide, exhibited cytotoxicity in older patients with MM [4,5,6]. The second-generation IMID, lenalidomide, revealed more potent cytotoxic potential than thalidomide and also reduced its toxicities. It is used as an induction therapy in combination with PIs plus dexamethasone as well as maintenance therapy with dexamethasone in patients with newly diagnosed MM (NDMM) or relapsed/refractory MM (RRMM) [4,5,6,18]. Furthermore, pomalidomide is about ten times more potent than lenalidomide and showed a clinical response in patients with RRMM who were refractory to lenalidomide or bortezomib. The third-generation IMiD of pomalidomide plus dexamethasone had shown a better response than these agents alone [4,5,6,18,19]. However, despite considerable recent advances in the treatment modalities of MM, the disease still remains incurable, and relapse is eventually inevitable for the majority of patients because of the heterogeneity, immune evasion, and persistence of drug-resistant clonal evolution, so it remains a serious issue [20,21,22]. Conventional therapies have shown limited benefits for high-risk patients because the outcome of patients with MM who were double refractory to PIs and IMiDs or relapsed after >3 prior lines of therapy is especially dismal. The 5-year overall survival (OS) rate is lower than 50% in MM patients with high-risk cytogenetics or frail elderly patients [4,5]. Therefore, effective treatments targeting novel pathways with minimal drug-toxicities and favorable tolerability as well as showing deep and durable responses are required.

Recently, cellular immunotherapies, directly targeting tumor-specific cell surface antigens, exhibiting more potent immune effector cells against tumor cells by redirecting or activating various effector-mediated killing of target cells, as well as revitalizing and reversing immunotolerance towards tumor cells, has become a rapidly evolving field for the treatment of MM [4,5,6,7,8,9,20,21,22]. The monoclonal antibodies (mAbs), daratumumab and elotuzumab, targeting CD38 and SLAMF7, respectively, were the first approved immunotherapies for MM in 2015 [23,24,25,26,27,28,29]. Afterward, the majority of clinical trials were conducted, with mAb monotherapy vs. combination therapy with mAb plus PIs or IMiDs, which also indicated promising results [29,30,31,32,33,34,35,36,37]. Additionally, efforts have been made to alter the administration routes as well as improve the constructs of mAb via structural modifications such as humanization, modification of Fc regions, the conjunction with small chemical molecules, or the addition of the second or the third specificity to recognize multiple distinct molecular targets. Thus, isatuximab, a novel humanized mAb targeting CD38, was developed, tested in clinical trials, and was just approved for use in 2020 [38,39,40,41,42,43,44]. More recently, a variety of novel immunotherapeutic approaches which act with different mechanisms have emerged to achieve deep and highly durable responses in MM, including bispecific or trispecific T cell engagers (BiTEs) and antibodies (duobody) [45,46,47], antibody drug conjugates (ADCs) [48], adoptive cellular therapy using dendritic cell vaccines, autologous chimeric antigen (CAR)-T cells or allogenic CAR-natural killer (NK) cells [49,50] and immune checkpoint blockade by immune checkpoint inhibitors [51]. These immunotherapies have been conducted in a variety of clinical trials with promising results except for checkpoint inhibitors, leading to the approval for the use of belantamab mafodotin; an afucosylated humanized IgG1 anti-B cell membrane antigen (BCMA) mAb conjugated with monometyl auristatin F (MMAF); a proteasome-resistant maleimidocaproyl linker to a microtubule-disrupting agent, for patients with RRMM that have been heavily pretreated with PI, IMiD, and anti-CD38mAb were intolerant to the mAb [52,53,54,55,56] in 2020. Moreover, the manageable safety profiles of belantamab mafodotin made it a potential candidate in combination regimens or adoptive cellular therapies targeting BCMA, which are currently examined in clinical trials.

Herein, this review provides an update on the most recent clinical trials and their relevant preclinical findings in novel cellular immunotherapeutic strategies against MM, especially ADCs, bi-specific or tri-specific T cell engagers or antibodies, autologous CAR-T cells, and allogenic CAR-NK cells, targeting BCMA or other novel targets and future perspectives.

## 2. Antibody Therapy

The development of antibodies in the treatment of cancers has been accelerating, and increasing evidence has shown that antibody therapies have marked efficacy and improve the outcome of patients with cancer [4,5,6,7,8,9,23,24,25,26,27,28,29,30,31,32,33,34,35,36,37,38,39,40,41,42,43,44,45,46,47,48]. Rituximab, obinutuzumab [57,58,59], brentuximab vedotin (BV) [60,61,62], blinatumomab [63,64,65], and inotuzumab ozogamicin [66,67,68,69] have already used in combination with chemotherapy regimens in malignant lymphoma or acute lymphoblastic leukemia. The development of immunotherapeutic modalities for MM has been delayed because of immune evasion by tumor cells through decreased expression of tumor-specific antigens, enhanced expression of inhibitory ligands such as programmed cell death ligand 1 (PD-L1) or major histocompatibility complex (MHC) molecules, and the recruitment of regulatory T cells (Tregs) or myeloid-derived suppressor cells (MDSCs), both of which work as immune suppressive cells [20,21,22,70,71,72,73,74]. The ideal tumor-specific antigen for immunotherapy would be a molecule that is uniformly and exclusively expressed on tumor cells and demonstrates a high efficacy but has low expression in normal tissues to avoid off-target adverse effects.

### 2.1. Monoclonal Antibodies (mAb) in MM

CD38, a 45-kDa type II transmembrane glycoprotein without an internal signaling domain, is widely and uniformly expressed on MM cells and is also expressed at a relatively low level on normal lymphoid cells, myeloid cells, and other non-hematopoietic tissues, providing the rationale for its use as a clinical target in MM [24]. CD38 retains multiple functions, including receptor-mediated regulation of cell adhesion, ectoenzyme activity, and signal transduction [24]. Humanized anti-CD38 mAbs, currently available for the treatment of MM, include intravenous daratumumab (DARA) [25,26,27,28,29,30,31,32,33,34,35,36,37], subcutaneous DARA [75], and isatuximab [38,39,40,41,42,43,44] (Figure 1).

DARA is a fully humanized IgG_1_ κ mAb targeting CD38, which kills MM cells through immune mechanisms of action, including complement-dependent cellular cytotoxicity (CDC), antibody-dependent cellular cytotoxicity (ADCC), and antibody-dependent cellular phagocytosis (ADCP) [25,26] (Table 1). Additionally, DARA induces direct programmed MM cell death; apoptosis through Fcγreceptor-mediated cross-linking, and the modulation of CD38 ectoenzyme function [27] (Table 1). Moreover, DARA has immunomodulatory effects through T cell activation and expansion, as well as the depletion of CD38+ Tregs, regulatory B cells (Bregs), and MDSCs [28]. It increases CD4+ and CD8+ T cells which enhance the host anti-tumor immune response against MM cells [28].

A phase 1/2 study and phase 2 study of DARA IV monotherapy in 148 RRMM patients treated with DARA 16 mg/kg, previously treated with >3 prior lines of therapy, showed an overall response rate (ORR) of 31.1%, including complete response (CR)+ stringent complete response (sCR) of 4.7% and very good partial response (VGPR) of 8.8%, a median progression-free survival (PFS) of 4.0 months and median overall survival (OS) of 20.1 months [27,28,29]. As a result, DARA IV monotherapy was approved in MM, relapsed after >1 prior line of therapy. In addition, DARA IV in combination with backbone regimens including IMiDs or PIs revealed deep and durable responses with significantly higher ORR and longer duration of response (DOR) [32,33,34,35,36,37]. Phase III POLLUX trials in 569 patients with RRMM demonstrated remarkable clinical activity and a significant benefit to a PFS by the treatment of DARA IV in combination with lenalidomide plus dexamethasone (DRd), compared with Rd, including an ORR of 92.9% (43.1% CR + sCR and 32.7% VGPR) vs. 76,4% (19.2% CR + sCR and 25.0% VGPR), a median PFS of not reached (NR) vs. 18.4 months, 12-month PFS rates of 83.2% vs. 60.1% and a 24-month PFS rate of 68.0% vs. 40.9%, restrictively [33,34]. A phase 3 MAIA clinical trial of DARA IV + Rd (DRd) (*n* = 368) vs Rd (*n* = 369) in 737 patients with newly diagnosed MM (NDMM) showed an ORR of 93% including 48% CR+sCR in the DRd group vs. 81% including 25% CR + sCR in the Rd group [75]. Both DOR and PFS were not yet reached with DRd group vs. 34.7 months and 31.9 months with Rd alone. The time to first response was 1.05 months in the DRd group, and the time to a CR or a better was 10.4 months vs, 11.2 months, respectively. After a median follow-up of 28 months, 70.6% of patients had not progressed with DRd vs. 55.6% of patients in the Rd group [75]. The EQUULEUS study of DARA IV in combination with pomalidomide plus dexamethasone (Pd) for 103 RRMM patients with previous >1 prior lines of therapy also showed deep and durable responses and were well tolerated with an ORR of 60%, including 17% CR + sCR and 25% VGPR, a median PFS of 8.8 months, 12-month PFS rate of 42%, median OS of 17.5 months and a median 12-month survival rate of 66% [34]. These encouraging clinical results led to the approval of a combination regimen of DARA+Rd (D-Rd) after >1 prior line of therapy in 2016 and DARA + Pd (D-Pd) after >2 prior lines of therapy in 2017, respectively, in relapsed MM. In addition, of note, the DRd regimen was approved in transplant-ineligible patients with newly diagnosed MM (NNMM) in 2019. A phase III Castor trials in 498 patients with RRMM showed a remarkable benefit to PFS in the treatment of DARA IV in combination with bortezomib plus dexamethasone, indicating an ORR of 82.9% (19.2% CR + sCR and 40% VGPR) vs. 63.2% (9% CR + sCR, 20.1% VGPR), a PFS of NR vs. 7.2 months, 12-month PFS rate of 60.7% vs. 26.9%, (time to progression) TTP at 12 months of 65% vs. 29% and a DOR of NR vs. 7.9 months [35,36].

Furthermore, updated Castor study demonstrated DARA-Vd (D-Vd) prolonged median PFS, compared with Vd in RRMM patients classified by cytogenetic risk (standard-risk group; 16.6 vs. 6.6 months, and a high-risk group; 12.6 vs. 6.2 months) [37], leading to the approval of D-Vd in MM relapsed after >1 prior line of therapy. Thus, based on the ALCYONE trial, the DARA-VMP (D-VMP) regimen was approved for transplant-ineligible NDMM patients [76,77]. Moreover, the D-Kd regimen was approved for patients with MM after 1–3 prior lines of therapy in 2020 [78].

Next, a phase 3 randomized, open-label, multicenter trial (COLUMBA), comparing the SC formulation of DARA vs. IV in 522 RRMM patients was conducted. It revealed that an ORR of DARA SC was similar with IV (41% vs. 37%, respectively), and DARA SC had a similar efficacy and safety profiles with DARA IV [79]. DARA SC pharmacokinetics were non-inferior to those o DARA IV. The majority of adverse effects (AEs) by the treatment of DARA SC occurred at the first injection. A nearly 3-fold reduction in systemic AEs was observed with DARA SC vs. DARA IV (13% including 2% of grade 3 vs. 34% including 5% of grade 3, respectively). DARA SC has a shorter administration duration of 3–5 min, so the infusion reaction rate (IRR) was significantly lower; thus, it increases treatment convenience for patients [79]. Similarly, a phase 2 multicohort, open-label trial (PIEIADES) evaluated the efficacy and safety of daratumumab SC in 132 patients with MM. Results demonstrated that 67 NDMM patients who were ineligible for SCT had an ORR of 88%, including 64% > VGPR in a combination regimen with VMP, and 65 patients relapsed or refractory after one or more prior lines of therapies achieved an ORR of 91% including 65% with >VGPR in combination therapy with D-Rd regimen [80]. Furthermore, the first phase 3 study of DARA SC combination therapy plus Pd in 304 RRMM patients with >1 prior line of therapy (APOLLO) showed that D-Pd had a significantly higher ORR rate, including CR + sCR and VGPR rates (69%, 25%, and 26% in the D-Pd group vs. 46%, 4% and 16% in the PD group, respectively) and minimal residual disease (MRD) negativity rate (9% vs. 2%, respectively) [81]. DARA SC + Pd regimen also significantly reduced the risk of progression or death by 37% vs. Pd (median PFS at a median follow-up of 16.9 months; 12.4 months vs. 6.9 months, 12-month PFS rate: 52% vs. 35%, respectively). Moreover, the D-Pd regimen achieved longer PFS in patients who were refractory to lenalidomide (9.9 months vs. 6.5 months, respectively) [81].

Isatuximab is a chimeric IgG_1_-κ anti-CD38 monoclonal antibody that binds to a specific epitope on the human CD38, targets different amino acid sequences from daratumumab, and elicits many cellular functions [38,39,40]. Isatuximab induces internalization of CD38 but not its significant release from the MM cell surface. In vitro analysis showed that continuous exposure to isatuximab does not decrease the level of CD38 on the surface of MM cells which contrasts with daratumumab [38,39]. It revealed not only ADCC, ADCP, and CDC but also strong direct programmed cell death; apoptosis independent of cross-linking and markedly inhibited ectoenzymic activity of CD38 in MM cells [40] (Table 1). Of note, isatuximab induces NK cell-mediated ADCC against MM cells independent of CD38 expression level on MM cells. In contrast, it revealed ADCP or CDC chiefly against highly CD38 expressed MM cells. Interestingly, although isatuximab activates NK lymphocytes, it paradoxically depletes NK lymphocytes. On the other hand, Tregs are not depleted by isatuximab [38,39].

Isatuximab monotherapy revealed an ORR of 23.8%, including 1 CR in 63 RRMM patients [41]. A phase 1b open-label, dose-escalation study of isatuximab plus Rd in 57 RRMM patients with a median of five prior lines of therapy (82% lenalidomide refractory) showed an ORR of 52%, overall median PFS of 8.5 months, and median DOR of 10.9 months in patients treated with isatuximab plus Rd among 42 evaluable lenalidomide-refractory patients [42]. Moreover, a phase 1b study of isatuximab plus Pd (Isa-Pd) in 45 RRMM patients with >2 prior lines of therapies (82% refractory to lenalidomide and 84% refractory to PIs) also revealed an ORR of 64.5% and a PFS of 17.6 months with a combination of isatuximab with Pd [43]. Moreover, in a phase 3 ICARIA-MM study in 307 patients with RRMM, responses were achieved faster and more durable in the IPd group, and the median time to first response in patients with >PR was 35 days in the IPd group vs. 58 days in the Pd group. The ORR was 57% including 5% CR + sCR, 27% VGPR and 29% PR in the IPd group was 38% including 1% CR + sCR, VGPR 7% and 27% PR in the Pd group. At a median follow-up of 11.6 months, median PFS was significantly longer in the IPd group (*n* = 154) compared with the Pd group (*n* = 153) (11.5 months vs. 6.5 months) [44].

CD26 is a 110-kDa transmembrane glycoprotein with dipeptidyl peptidase (DPPIV) activity, which is involved in T cell activation and tumorigenesis. We identified that CD26 is expressed in normal human osteoclasts and was intensely expressed in activated osteoclasts in osteolytic bone tumors, including MM [82]. In addition, CD26 was expressed in plasma cells around osteoclasts or endothelial cells in the BM tissues of several MM patients [83]. We demonstrated that a humanized anti-CD26 IgG_1_ mAb inhibited human OC differentiation in in vitro and in vivo studies [82,83]. Anti-CD26mAb enhanced cytotoxicity against CD26 positive MM cells, chiefly via ADCC, direct effects and inhibition of the adhesion between MM cells and their microenvironment such as BM stromal cells [83]. Moreover, anti-CD26mAb in combination with PI or IMiD synergistically enhanced mAb-induced ADCC activity against CD26 positive MM cells, compared with monotherapy. Eventually, anti-CD26mAb significantly reduced MM cell burden and osteoclast formation using an intra-bone tumor xenograft model of MM. Our preclinical results strongly suggested that CD26 might be an attractive therapeutic target of novel mAb therapy in MM [82,83].

### 2.2. Bi-Specific Antibodies (BsAbs) and Tri-Specific Antibodies (TriAbs) in MM

**Figure 2:**
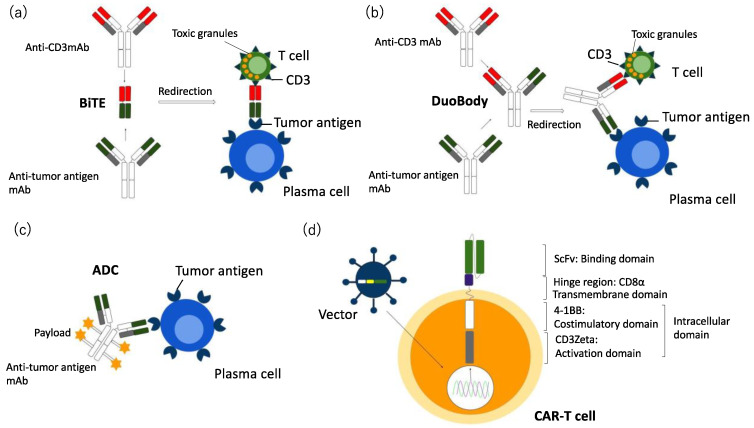
A diagram of structure. A bispecific antibody; (**a**) bispecific T cell engager (BiTE), (**b**) duobody construct, (**c**) an antibody-drug conjugate (ADC), and (**d**) a chimeric antigen receptor (CAR)-T cell.

Bi-specific antibodies (BsAbs) concomitantly bind to two different antigens; one side binds to CD3 on T cells or CD16 on NK cells, and the other side binds to a tumor-specific antigen on tumor cells, which redirects the host immune system for cytotoxic T cell activation to secrete cytotoxic granules such as granzyme B, perforin and interferon, leading to exhibit effector cell-mediated cytotoxicity against tumor cells [45,46,47]. To date, multiple BsAbs, including the bispecific T cell engager (BiTE) and the duobody antibody have been developed and tested in numerous clinical trials (Figure 2a,b). The BiTE, which consists of double antigen-binding sites including double single-chain variable fragments (scFv) from two antibodies, without containing Fc regions, are connected by a flexible linker and redirects effector cells to lyse targeted tumor cells (Figure 2a). The duobody construct comprised a single IgG_1_ heterodimeric BsAb, generated via the fusion of heavy and light chain homodimers derived from two different antibodies, using Fab-arm exchange (Figure 2b) [45,46,47,84,85,86]. The BiTE represents a small molecule targeting more than one antigen with a single antibody, which makes it possible to add other molecules to create tri-specific antibodies (TrisAbs). It is engineered to have three antigen-binding sites to bind to tumor-specific antigens on tumor cells, CD3; a T cell receptor (TCR) on T cells, and CD28; co-stimulatory molecules, also expressed on T cells. The costimulatory signal by CD28 not only promotes cytotoxic T cell activation and proliferation, but the binding of TrisAb to CD28 on myeloma cells also increases the affinity of Ab to tumor cells, which leads to reveal higher cytotoxic potential. Multivalent BsAb targeting multiple tumor antigens in a single antibody, BsAb with modified Fc regions and bispecific NK cell engager (BiKEs) or antibody which redirect NK cells to reveal increased FcR-mediated cytotoxicity against tumor cells, are also effective to overcome refractory disease to available therapies because of immune evasion by the down-regulation or heterogeneous expression of tumor-specific antigens in tumor cells [45,46,47,84,85,86,87]. Moreover, BsAbs also redirect cytotoxic T cells to activate normal T cells, which lead to occur therapy-related AEs in normal tissues, including hematological abnormalities, cytokine release syndrome (CRS), neurotoxicity, and multi-organ failure [84,85,86]. Thus, as an alternative approach to overcome these AE, BiKEs or trispecific NK cell engagers (TriKEs), and antibodies are effective in reducing these off-target toxicities, including leukopenia and lymphopenia and its associated infections. NK cell redirecting is also effective for the eradication of MRD in MM [45,46,47].

Blinatumomab, a BiTE composed of a 55-kDa fusion protein that contains scFvs, linked by a glycine-serine linker, targets CD19 on tumor cells by the redirection of CD3 positive cytotoxic T cells to lyse CD19 positive tumor cells. It resulted in a significantly longer OS, compared with chemotherapy alone in patients with relapsed or refractory B cell precursor, ALL, and is currently available [63,64,65].

B cell maturation antigen (BCMA), type III transmembrane receptor which belongs to tumor necrosis factor superfamily member 17, known as TNFRSF17/CD269, is one of the most attractive antigens in the development of immunotherapies of MM, because it is exclusively expressed in most plasma cells, compared with CD38 and SLAMF7. BCMA binds to its ligands, B cell activating factor (BAFF) and a proliferation-inducing ligand (APRIL), and is involved in the late stage B cell differentiation. Indeed, BCMA leads to differentiate plasma cells with its increased expression and promotes the survival of plasma cells [47,48,84,85,86]. BCMA is highly expressed on plasma cells in MM patients, compared with normal plasma cells, so it may be a biomarker for diagnosis or predicting treatment response in MM. BCMA is down-regulated from the surface of plasma cells by *γ* secretase-mediated cleavage, resulting in the production of a soluble form BCMA (sBCMA), which causes a decrease in its expression on tumor cells. An increased level of sBCMA in the serum is also related to a high tumor burden and worse prognosis in MM [84,85,86]. Therefore, a decrease of sBCMA, observed in patients suggests a good treatment response and indicates sBCMA as a novel prognostic biomarker in MM [84,85,86].

AMG420 (BI-836909) is a novel BiTE that targets both CD3 on T cells and BCMA on MM cells concomitantly to induce MM cell lysis via the cytokine release, such as perforin and granzyme B by activated T cells [87,88]. AMG420 demonstrated its cytotoxic efficacy in preclinical xenograft models [87]. A first-in-human, phase I dose-escalation study in 43 RRMM patients with a median 3.5 prior lines of therapy exhibited 31% of an ORR. At the maximum dose of 400 μg/day, 70% of patients achieved an ORR, including 57% sCR+CR, and 50% of patients had MRD negativity. [88]. AEs showed that 38% of patients had CRS including 2% > grade 3 and 33% had infections, including 24% > grade 3. Neurotoxicity (grade > 3) occurred in 7% of patients [88]. In addition, several other novel BCMA/CD3 BiTEs have been developed, and clinical trials have been conducted or are currently underway (Table 2).

AMG701 is an anti-BCMA × CD3 half-life extended BiTE, composed of two scFVs and Fc regions. A phase 1 study of AMG 701 in 75 patients with RRMM or intolerant with a median prior six lines of therapy showed an ORR of 36% at doses of 3–12 mg, time to best response of 2.8 months, and DOR of 3.8 months [89]. Hematological AEs include anemia (40%), neutropenia (23%), thrombocytopenia (20%), infections (39%), and CRS (63%), including 7% with a grade > 3, which were resolved with corticosteroid or tocilizumab with a median duration of two days. Neurotoxicity occurred in 7% of patients with grade 1 with a median duration of one day [89]. This study of AMG701 in RRMM patients is currently in progress and is expected to end in 2026 (Table 2).

Teclistamab (JNJ-64007957) is a humanized BCMA × CD3 IgG_4_ duobody BsAb which was shown to induce T cell-mediated killing of MM cells in heavily pretreated patients and in xenograft models [90,91]. A phase 1 study of tesclistamab, administrated via IV (*n* = 84) or SC (*ssc* = 65) in 149 RRMM patients with a median of six prior lines of therapy, showed a deep and durable response with a manageable safety profile at high dose levels. The most effective doses of teclistamab were IV (270–720 μg/kg weekly) and SC (720–3000 μg/kg) and revealed an ORR of 69% (67% vs. 71%, respectively), including 59% > VGPR and 26% CR. Among 11 evaluable patients, eight (72%) patients attained MRD-neg CR at 10^−5^ and 1 (9%) at 10^−6^. Moreover, tecilstamab at the recommended phase II dose (RP2D) of 500 μg/kg SC revealed an ORR of 73%, including 73% > VGPR and 23% > CR and PFS of 94% at a median 3.9-month follow-up [92]. Most common AEs include hematological abnormalities, such as anemia (55%), neutropenia (57%), and thrombocytopenia (22%). Non-hematological abnormalities include CRS (55%) with no > grade 3 events, infections (52%) including 15% > grade 3, injection site reaction (32%), and neurotoxicity (5%), including 2% > grade 3. CRS occurred in 54% in the IV cohort and 57% in the SC cohort with a median time to onset of two days and a median duration of two days, which were treated with tocilizumab (23%), and a steroid (13%), or a vasopressor (1%). Phase 2 clinical trial of teclistamab monotherapy at the dose level of 1500 μg/kg is planned [92] (Table 2).

REGN5458, also known as a BCMA × CD3 BsAb, also induced a deep and durable response with time as well as an early response in patients with RRMM [93]. A phase 1 study of REGN in 49 patients who had a median of five prior lines of therapy showed an ORR of 35.6%, including 62.5% at the active dose level of 60 mg. Among responders, 95% had > VGPR and 42% had CR + sCR. In addition, 57% of patients achieved MRD negativity at 10^−5^ in evaluable patients [94]. Of note, these responses were not correlated with BCMA expression in MM cells. Hematological AEs were revealed in > 15% of patients, including anemia (37%), lymphopenia (18%), thrombocytopenia (18%), and neutropenia (13%). Non-hematological AEs include infections (47%, 18% with grade > 3), CRS (39%), fatigue (35%), nausea (31%), and pyrexia (31%). The CRS onset commonly within the first week of treatments at the initial doses, and the median duration of CRS was 11.7 h. Eighty-four percent of CRS cases were grade 1 without any grade 3 cases. Fifty-three percent of patients needed supportive treatments to treat CRS with tocilizumab (12%) or corticosteroids (21%). Twelve percent of patients experienced neurotoxicity with grade 1 or 2. Patients’ condition became improved within four weeks and was maintained. A median DOR was six months, and 37% of responders maintained a response for >8 months and continued to undergo the therapy [93] (Table 2).

TNB-383B is a unique fully-human triple-chain BCMA × CD3 BsAb which is designed to have a unique αCD3 moiety that lyses target cells with minimal cytokine release, two αBCMA domains which favors cell surface BCMA binding and silenced IgG_4_ backbone to prevent nonspecific T cell activation [94]. In vitro and in vivo studies have demonstrated its activity without toxic cytokine secretion. A phase 1 study of TNB-383B in 58 RRMM patients with a median of six prior lines of therapy showed an ORR of 80% at dose levels >40 mg of TNB-383B, including 13.3% CR + sCR, 60% VGPR, and 75% of MRD negativity at10^−5^–10^−6^ among evaluable patients. 81% of patients continue to maintain response for median 18 weeks up to 39 weeks, and responses continued to deepen [94]. As a convenience, TNB-383B was administrated into patients as a bolus at all dose levels without requiring step-slit-dosing. Most common AEs included hematological toxicities; anemia (21%, 17% > grade 3), thrombocytopenia (17%, 14% > grade 3), neutropenia (19%, 16% > grade 3) and infection (21%, 14% > grade 3). Non-hematological AEs included CRS (45%) without > grade 3. The median onset of CRS was on day one, and the median duration of CRS was one day. The number of patients whose condition of CRS became worse with an increased dose level of TNB 383-B was minimal. Tocilizumab was used only in <5% of CRS patients. No other remarkable increase in the incidence of AEs excluding CRS was observed at a higher dose level of TNB383-B [94]. A continuous study is currently ongoing (Table 2).

GPRC5D: G Protein-Coupled Receptor Class C Group 5 Member D is highly expressed in plasma cells of MM, but limited expression was also detected in normal human tissues such as normal plasma cells and hair follicles [95,96]; shed peptides, or extracellular domain shedding is not known. The expression of GPRC5D may be an ideal target for CD3 redirection in MM, and it may be predictive of poor prognosis factor. Anti-tumor activity was demonstrated in xenograft models of MM [95]. Talquetamab (JNJ-64407564) is a first-in-class duobody IgG_4_ PAA antibody that binds to GPRC5D and CD3 to redirect T cells for T cell mediated lysis of GPRC5D positive MM cells [95]. A phase 1 study of talquetamab in 155 RRMM patients showed encouraging, durable, and deep responses with an ORR of 63% (67% vs. 66%, in an IV cohort vs. an SC cohort, respectively), including 42% > VGPR across all doses [96]. In addition, an ORR of 69% at doses of 450 μg/kg SC (RP2D), including >39%VGPR was achieved. Of note, 67% of triple-class refractory patients, as well as 100% of penta-drug refractory patients, responded to the therapy within one month during the median duration of 3.7 months. None of the responders at doses > 405 μg/kg SC progressed within this period, and 81% of patients continued to maintain responses for up to a median of 18 weeks [96]. Manageable safety profiles were also shown. Common AEs were hematologic abnormalities including anemia (48%), neutropenia (47%), lymphopenia (40%), leukopenia (32%), and thrombocytopenia (32%). Non-hematological AEs included CRS (54%, 48% vs. 64%, respectively), skin disorders (45%), infection (38%), dysgeusia (36%), pyrexia (27%). and headache (27%). CRS occurred in 68% of patients who received talquetamab at the dose of 405 μg/kg SC, including 10% > grade 3. The median time to CRS onset was two days and the median duration of CRS was two days. Eighty-one percent of patients received supportive treatments such as tocilizumab (40%), corticosteroids (8%), oxygen (8%), or vasopressors (2%). Neurotoxicity was also seen in 6% of patients in IV cohorts, including 2% grade > 3. Grade > 3 CRS or other neurotoxicity did not occur with SC dosing cohorts [96] (Table 2).

Fc receptor-homolog 5 (FcRH5) is a type I membrane protein that is expressed in normal plasma cells and B cells and is more intensely expressed on MM cells [96]. Cevostamab (BFCR4350A) is a humanized IgG-based FcRH5 × CD3 T cell engaging BsAb which leads to enhance the killing of MM cells. A phase 1 clinical study of cevostatmab monotherapy was performed to evaluate its safety and activity in patients with 53RRMM with a median of six prior lines of therapy. It showed a highly active and deep as well as durable response with an ORR of 53% in patients treated with active doses of > 3.6 mg/20 mg (41% in penta-drug-refractory patients and 63% in patients with prior anti-BCMA therapy), including 18% sCR + CR and 15% VGPR. In addition, MRD negativity at 10^−^^5^ was achieved in 85% of evaluable patients with >VGPR. The median time to first response was 29.5 days, and eight patients showed a median DOR of six months. Four patients continued to respond even when the treatment was discontinued [97]. Treatment-related toxicities were safely manageable with a single step-up dosing schedule in the first cycle to reduce the risk of severity in the development of CRS. Notwithstanding, 53% of patients experienced serious AEs. Hematological AEs revealed thrombocytopenia (28%), anemia (28%), neutropenia (17%), and leukopenia (15%), while non-hematological AEs included CRS (76%) at grade 1 or 2 (74%) or grade 3 (2%), and other neurotoxicity (>2%). A median time to CRS onset was 6–12 h, and 66% of CRS occurred at the first cycle, and all CRS events resolved with the treatment of tocilizumab (25%) or steroids (17%). A dose-escalation and expansion clinical study is expected to be planned [97] (Table 2).

CC-93629 is a humanized IgG trivalent BCMA × CD3 BsAb. A phase 1 clinical trial of CC93629 in 19 patients with RRMM and a median of six prior lines of therapy showed an ORR of 36% in the 3–6 mg cohort and 89% in the >6 mg cohort, including 17% CR. Ninety-two percent of patients achieved MRD negativity and the median time to response was 4.1 weeks. Ninety-seven percent of patients had AEs, including CRS, which occurred in 77% of patients, with most cases being grade 1 or 2 [98] (Table 2).

PF0683135 is a humanized BCMA × CD3 BsAb. A phase 1 clinical trial of PF0683135 in 18 patients with RRMM and a median of seven prior lines of therapies, including anti-BCMA therapy (29%), demonstrated an ORR of 75% at the doses of 215 and 260 μg/kg. CRS with grade 1 or 2 occurred in 61% of patients [99] (Table 2).

Moreover, CD3 × CD38 BiTE antibody, engineered to direct T cells to CD38 on tumor cells, was also developed. Phase 1 multicenter study of GBR1342 and AMG424 is currently in progress and planned to be continued until 2021 or 2022. [100,101] (Table 2).

### 2.3. Antibody Drug Conjugate (ADC) in MM

The ADCs consist of three fundamental elements: a tumor-specific mAb including an antigen-binding domain and an Fc portion, and a payload; a cytotoxic drug and non-cleavable linker which connects mAb with payload [48,84,85,86] (Figure 2c). The payload: antibody ratio (DAR) varies between ADCs, which affects cytotoxic activity, stability, and immunogenicity. After binding to a tumor-specific antigen-binding site on tumor cells, the conjugate complex undergoes receptor-mediated endocytosis and is trafficked to the lysosome. Then, in the lysosome, the linker is cleaved, and payloads are released intracellularly, leading to cytotoxicity against tumor cells. ADCs target tumor cells while sparing normal cells, thereby maximizing their cytotoxic efficacy but minimizing systemic toxicity [48,84,85,86].

Brentuximab bedotin (BV; Adsetris^R^, SGN-35) is an ADC comprising an anti-CD30 mAb conjugated by a protease cleavable linker to the potent anti-microtube agent, monomethyl auristatin E (MMAE), which was developed and approved for relapsed or refractory Hodgkin’s lymphoma (HL), cutaneous T cell lymphoma (CTCL) and anaplastic large T cell lymphoma (ALCL) [60,61,62]. The payload consisting of MMAE binds to the tubulin and disrupts the microtube network, and induces cell cycle arrest, leading to the death of CD30 positive lymphoma cells. In relapsed or refractory HL or CD30+ CTCL, BV is well-tolerated, and the ORR was significantly improved in BV-treated patients [60,61,62]. Inotuzumab ozogamicin (Besponsa^R^ or CMC-544) is also an ADC, comprised of a humanized anti-CD22 mAb conjugated to calicheamicin. It binds to the minor groove of DNA and induces double-strand cleavage, leading to subsequent apoptosis in tumor cells [66,67,68,69]. The ADC showed both high ORR and MRD negativity which improved the PFS and OS in patients with relapsed or refractory acute lymphoblastic leukemia (ALL), compared with standard chemotherapy alone [66]. In addition, the ADC incorporated into chemotherapy regimens was safe and effective as a salvage or front-line therapy in elderly ALL patients [66,67,68,69].

Belantamab mafodotin (GSK2857916), the first humanized anti-BMCA IgG_1_ ADC, was approved for MM with >4 prior lines of treatment including IMiDs, consists of the antibody component of an afucosylated IgG1 directed against BCMA mAb and the small molecule of the tubulin polymeraization inhibitor, MMAF via a non-cleavable linker [52]. Through binding to BCMA, Belantamab undergoes its internalization, followed by the release of MMAF via proteolytic cleavage. The afucosylated Fc portion has the benefit of promoting the binding of effector cells to BCMA positive MM cells and augment effector-mediated cytotoxicity by ADCC and ADCP as well as MMAF-induced apoptosis against MM cells [52]. In MM xenograft models, GSK2857916 depleted MM cells but not BCMA- BM microenvironmental cells [52]. A multicenter phase 1 trial of GSK2857916 in 35 patients with RRMM after autologous stem cell transplantation (ASCT) (DREAMM-1) showed an ORR of 60% with 14% CR and 60% VGPR. The median PFS and DOR were 12 and 14.3 months, respectively [53]. It also revealed a manageable and safety profile [53,54,55] (Table 3).

Additionally, a phase 2 study of belantamab mafodotin showed clinically important results for patients with RRMM (DREAMM-2). One hundred and ninety-six patients were randomized to receive belantamab mafodotin at the dose of 2.5 mg/kg or 3.4 mg/kg and achieved an ORR of 34% (3.4 mg/kg cohort) and 31% (2.5 mg/kg cohort), including 19% and 20% > VGPR, respectively. The PFS was 2.9 months (2.5 mg/kg cohort) and 4.9 months (3.4 mg/kg cohort), and the median DOR was not reached in both groups [56]. Therapy-related AEs were detected in the majority of patients (98% and 100%, respectively), and dose modifications including dose delay (54% and 62%, respectively) or dose reduction (29% and 41%, respectively) were needed (Table 3).

The most common AEs across all doses were eye disorders, including keratopathy (71%, 44% > grade 3), decreased visual acuity (53%, 28% > grade 3), blurred vision (22%, 4% > grade 3), and dry eye (14%, 1% > grade 3). The median time to the onset of keratopathy was similar regardless of the use of corticosteroid eye drops (24 days and 25 days in corticosteroid eye drop-treated vs. 27 days and 25 days in not treated) [56]. Dose modifications for corneal AEs are based on both corneal epithelium change observed by ophthalmic examinations and changes in best corrected visual acuity (BCVA). Although cases of corneal symptoms are not very frequent, visual changes are more likely to be symptomatic, suggesting its diagnosis. Indeed, the mechanism for cornea events was uncertain, and micropinocytosis-dependent internalization of ADCs into the cornea or target organs may result in off-target toxicity, which was caused by the linker-payload, but not the target cells in the cornea or the antibody itself [102]. Another major AEs across all doses include hematological abnormalities including thrombocytopenia (62%, 21% > grade 3), leukopenia (49%, 21% > grade 3), anemia (32%, 18% > grade 3) and neutropenia (28%, 9% > grade 3), infusion reactions (21%, 3% > grade 3), and gastrointestinal symptoms [56]. Altogether, belantamab mafodotin retained a manageable safety profile and has the potential to have anti-MM activity in RRMM [53,54,55,56].

A randomized phase II/II trial comparing belantamab mafodotin monotherapy with combination therapy with pembrolizumab (DREAMM-4), lenalidomide plus dexamethasone (arm A, DREAMM-6) or bortezomib plus dexamethasone (arm B, DREAMM-6) revealed encouraging clinical activities in heavily pretreated MM patients, indicating a potential candidate of the ADC for use incorporated into other treatment regimens. Blenrep^R^ was the first approved anti-BCMA ADC for use in the treatment of MM in 2020 (Figure 1). Currently, several clinical trials of the ADC are in progress or planned in transplant-ineligible patients with newly diagnosed MM patients [48] (Table 3).

Other ADCs targeting BCMA include MEDI2228, AMG224, and CC99712 [103,104,105] (Table 3). MEDI2228 is a fully human ADC conjugated to a DNA cross-linking pyrrolobenzodiazepine (PBD) dimer through a protease-cleavable linker, which targets the extracellular domain of BCMA binding to the membrane rather than circulating soluble BCMA [103]. Once the ADC is internalized, it is cleaved and trafficked into the lysosome and releases PBD, which conducts cross-linking with DNA, leading to cause apoptotic death of tumor cells. A phase 1 clinical trial of MEDI2228 in 82 RRMM patients, heavily pretreated with PI, IMiD, and anti-CD38mAb therapy revealed an ORR of 65.9%, including 2.4% CR + sCR and 24.4% VGPR and a median DOR of 5.9 months at the dose of 0.14 mg/kg Q3W cohort and also showed its manageable safety profile. AEs include thrombocytopenia (31.7%, 24.4% > grade 3), photophobia (58.5%, 17.1% > grade 3) and dry eye (20%), skin eruption (31.7%, 0% > grade 3), and pleural effusion (24.4%, 2% > grade 3). Early-onset photophobia was reversible after drug discontinuation and was improved over time [103]. Further studies with an ongoing cohort of 0.14 mg/kg are needed to explore alternative dose schedules to prevent eye toxicities (Table 3).

Clinical trials of ADCs targeting molecules for the treatment of MM, other than BCMA were also conducted or are currently underway using a humanized anti-CD38mAb conjugated to Shiga like toxin (TAK-169) or IFNα2b (TAK-571), indatuximab ravtansine (BT062); a chimeric B-B4, an afucosylated anti-CD138 IgG_4_ mAb conjugated to ravtansine (DM4) via a SPDB dislufide cleavable linker [106,107], lorvotuzumab mertanisine (IMGN901); a humanized anti-CD56 IgG1 mAb conjugated to mertansine (DM1) via a stable disulfide linker [108,109], dacetuzumab, lucatumumab, milatuzumab doxorubicin (STRO-001); a humanized anti-CD74mAb conjugated to doxorubicin via an acid-labile hydrazone linker [110,111] and FOR46; MMAE-conjugated anti-CD46mAb (Table 3). Furthermore, a bispecific ADC, consisting of two tumor binding sites linked to cytotoxic payloads has been developed. It will have the potential to reveal benefits for patients in the future.

## 3. Chimeric Antigen Receptor (CAR) T Cell Therapy in MM

Autologous cytotoxic T cells have a high potential to eradicate tumor cells by recognizing tumor-specific antigens in cancer. CARs are artificial fusion proteins that consist of extracellular antigen recognition and binding domain (scFvs), hinge; a modified spacer, CD28 or CD8, which redirects CAR-T cells to recognize tumor cells, intracellular signaling domain which activates T cells and transmembrane domains which connect the antigen-binding domain to the signaling domain [49,50,112,113,114] (Figure 2d). In addition, an intracellular signaling domain comprises the T cell activation domain; CD3Zeta, and one or two co-stimulatory domains; 4-1BB (CD137), CD28, CD27, or OX40-1. First-generation CARs contained only CD3Zeta in its co-stimulatory domain, so its signal was too weak to elicit enough anti-tumor activity. Thereby, second-generation CARs, which retained chiefly 4-1BB or CD28, essential for T cell function, metabolism, and proliferation as a co-stimulatory signal, were developed. These second-generation CAR-T cells with CD28 co-stimulatory molecules differentiate into effector memory T cells, and those containing 4-1BB domains differentiate into central memory T cells, both of which were able to proliferate more effectively and enhance expansion after tumor-antigen exposure [49,50,112,113,114] and produce an increased level of cytokines such as perforin or granzyme B, leading to higher anti-tumor activity in preclinical models.

CAR-T cell therapy is a novel cellular immunotherapy that combines the advantage of the target specificity of mAbs and the cytotoxicity of T cells. In contrast to human leukocyte antigen (HLA)-restricted TCRs, CARs are not HLA-restricted; therefore, patients of any HLA type can be treated with CAR-T cells [49]. Indeed, multiple manufacturing processes are involved before CAR-T cells are administrated into patients [49,50,112,113,114,115]. The first step of generating CAR- T cells begins with leukapheresis of peripheral blood to obtain CD3+ T cells from patients for autologous CAR-T cells or healthy donors for allogenic CAR-T cells. Collected leukocytes undergo an engineering process to express CD3 and CD28 or 4-1BB using beads coated with each mAb. These cells are subsequently expanded in culture and activated. Second, activated T cells, prepared at the first step, are transduced with a vector carrying a gene, encoding a receptor to the tumor-specific antigen on tumor cells, and the cells are genetically modified to express CAR genes. Afterward, CAR-expressing T cells are expanded and formulated. The manufacturing process takes at least two to four weeks. Therefore, patients may have to undergo bridging chemotherapy to control underlying disease conditions. In addition, prior to the infusion of CAR-T cells, most patients have to receive a conditioning lymphodepleting chemotherapy consisting of fludarabine plus cyclophosphamide to prepare the environment for the proliferation of infused CAR-T cells [49,50,112,113,114,115]. Once administrated into patients, CAR-T cells encounter the targeted-antigen, expand, and the kinetics of CAR-T cell expansion peak after CAR-T cell infusion within seven days. Thus, the cells eventually recognize, kill targeted tumor cells and prolong their persistence [49,50,112,113,114,115].

The therapeutic efficacies of CAR-T cells in patients with relapsed or refractory B cell malignancies who have only limited treatment options have shown to be promising in multiple clinical trials [115,116,117,118,119,120,121,122,123,124,125,126,127,128]. Autologous CD19-targeted CAR-T cells were approved for clinical use in patients with B-acute lymphoblastic leukemia (ALL) or diffuse large B cell lymphoma (DLBCL) in 2017 [116,117,118,119,120,121,122,123]. These patients revealed the most striking responses by the infusion of CD19-targeted CAR-T cells; tisagenlecleucel (Tisa-cel) or axicabtagene ciloleucel (Axi-cel) [116,117,118,119,120,121,122]. However, CD19-targeted CAR-T cell therapy was not approved for the treatment of MM because CD19 is rarely expressed in MM cells. Among 10 patients with RRMM who received CD19 CAR-T cell (CTL019) infusion after a second ASCT, because of poor treatment response after the first ASCT, two patients had prolonged PFS, implying benefit of CTL019 following ASCT [124]. Large-scale clinical trials to investigate the efficacy of anti-CD19 CAR-T cell therapy in the majority of patients who have RRMM are crucial to validate the results.

CAR-T cell therapy induces unique on-target and off-tumor toxicities, including CRS and neurotoxicities such as immune effector cell-associated neurotoxicity syndrome (ICANS), some of which can be life-threatening [129,130]. CRS is also observed in immunotherapies by mAb, ADC, and BiTE. In addition, ICANS can be caused by BiTE therapy. The common feature of CRS is signs of systemic inflammation, including fever, hypotension, tachycardia, hypoxia, and rigor, which are observed via the release of multiple cytokines or chemokines such as IL-6 or IFN γproduced by CAR-T cells or immune effector T cells. Most cases usually become reversible and shortly resolved with the treatment of immunosuppressive agents, including IL-6 antagonists; tocilizumab or corticosteroid [114,115,116,117,118,119,120,121,122,123,124,125,126,127,128,129,130,131]. While high-grade (grade 3 or 4) ICANS sometimes require prolonged hospitalization and intensive supportive care in a subset of patients whose recovery is delayed [129,130,131]. The symptom of ICANS includes delirium, confusion, headache, aphasia, consciousness, somnolence, tremors, seizures, ataxia, and dysarthria [129,130,131]. Nonetheless, MRI abnormalities are detected in only 30% of patients treated with CAR-T cells. Cerebral edema is the most common finding of MRI in patients diagnosed as ICANS, followed by leptomeningeal enhancement and multifocal microhemorrhage [130]. Deng et al. reported the ratio of small populations of monocyte-like ICANS-associated cells, termed IACs, included in CAR-T cell products at the initial infusion was associated with the development of high-grade ICANS [132]. The IACs were identified to reveal the high expression level of 257 genes, including IL-1B, IL-8 (CXCL8), CD68, LYZ, SPI1 LIRB4, and SIRPA, compared with other cells, except for IACs [132]. In brief, heterogeneity and diversity of cellular and molecular features in CAR-T cells might contribute to induce variable toxicities by CAR-T cell therapy. Although the pathogenesis of ICANS has not been clearly elucidated yet, severe cases of ICANS exclusively occur after the development of CRS, and several factors, including high tumor-burden or a higher peak of CAR-T cells by in vivo expansion, were reported to be involved [130,131].

In CAR-T cell therapy, it is important to attain an increased anti-tumor efficacy with decreased on-target and off-target toxicities. Deng et al. showed that the early molecular response (EMR) during the first week after CAR-T cell infusion was correlated with clinical efficacy evaluated by PET/CT at three months, and poor EMR was associated with CD8 T cell exhaustion included in CAR-T cells [132]. Therefore, inhibitory blocking antibodies to target exhaustion markers such as TIM3, LAG3, or PD-1 in CD8 T cells might improve efficacy in patients with poor EMR after CAR-T cell infusion [132].

Moreover, to reduce on-target and off-tumor toxicities, it is ideal to target antigens, that are absent or genetically modified to remove them from normal cells. For instance, CAR-T cell therapy in AML is limited because cell surface antigens expressed on leukemia cells such as CD33 are concomitantly present on normal cells. This could be overcome by clustered regulatory interspaced short palindromic repeats (CRISPER)/CRISPER-associated protein 9 (CRISPER/Cas 9) gene modification techniques, which integrated a targeted gene into specific sites of the genome. Namely, using CRISPER/Cas9 techniques, anti-CD33 targeting CAR-T cells could target only CD33 positive leukemic cells and not normal cells via the removal of CD33 in normal hematopoietic progenitor cells (HPCs). Thus, these CAR-T cells administrated into mice with AML were capable of killing only CD33 positive leukemic cells and not normal myeloid HPCs without the occurrence of cytopenia as an off-target toxicity [133].

Currently, efforts are focused on identifying novel targets other than BCMA, widely and exclusively expressed on MM cells but not normal cells for the treatment of MM. Indeed, various CAR-T cell products have been evaluated targeting CD19, CD38, CD138, SLAMF7, Immunoglobulin κ, CD56, CD44v6, CD70, CD138, BAFF, and integrin β7 in pre-clinical studies or phase I/II clinical trials for heavily-treated patients with RRMM [123,125,126,127,128,134,135,136,137,138,139,140,141,142,143,144,145] (Table 4).

BCMA is specifically expressed on MM cells in the majority of MM patients and may also be a promising antigen target for CAR-T cell therapy in MM. BCMA-targeted CAR-T cells, bb2121 (Ide-cel), which contains a second-generation CAR incorporating an anti-BCMA single-chain variable fragment with 4-1BB co-stimulatory domain and CD3Zeta signaling domain, revealed potent cytotoxicity against MM cells regardless of the ratio of BCMA expression in MM cells or the presence of soluble BCMA [123]. A multicenter phase 1 study of bb2121 in 33 patients with RRMM who had received >3 prior lines of therapy revealed promising clinical results (CRB-401) [123,146]. The ORR was 85%, including 45% of patients with 9% CR or 36% sCR, and the median PFS was 11.8 months with 40% of the patients free of progression at 12 months. Ninety-five percent of patients who had a response with PR or VGPR had MRD negativity at 10^−5^. The safety profile of bb2121 to attain CR was observed at doses from 150 × 10^6^ to 800 × 10^6^ CAR-T cells. Patients who received 450 × 10^6^ CAR-T cells attained a similar response rate, independent of tumor BCMA expression levels of less than 50% or more than 50% in plasma cells (100% vs. 91%, respectively). Common AEs included hematological abnormalities including neutropenia (92%), leukopenia (61%), anemia (58%), and thrombocytopenia (58%) [123]. Neurotoxicity including ICANS occurred in 42% and CRS in 76% of patients. The severity of most CRS events was grade 1 or 2 (only 7% > grade 3), which was treated with tocilizumab or glucocorticoid successfully [123]. Thus, anti-BCMA CAR-T cell therapy could be performed safely and effectively in heavily pretreated MM patients, so it is expected to obtain approval in the near future. Furthermore, clinical trials with a large number of patients and longer follow-up are required to evaluate whether this therapy contribute to the cure of MM (Table 5).

The phase 1 CRB402 (bb21217) study of anti-BCMA CAR-T cells in 74 RRMM patients who received >3 prior lines of therapy revealed an ORR of 55%, including 18% CR and 30% VGPR. The median time to CR was 2.5 months, and the median DOR was 11.9 months at the dose level of 150–450 × 10^6^ CAR-T cells [147] (Table 5).

CT053 was also transduced second-generation CAR utilizing a fully human BCMA-specific scFv. A phase 1 study of 24 patients with RRMM who had received >2 prior lines of therapy showed an early, deep, and durable response; an ORR of 87.5%, including 79.2% with 12.5% CR or 66.7% sCR, and a DOR of 21.8 months [148]. The PFS was 18.8 months with 6- and 12-month PFS rates of 87% and 60.9%, respectively. Phase 1b/2 Lummicar-2 study of 20 RRMM patients, 14 patients received CT053 infusions at a dose of 1.5–1.8 × 10^8^/kg in six patients, and 2.5 × 3.0 × 10^8^/kg in eight patients, and a deep and durable high response was seen. The results showed an ORR of 100%, including 40% CR + sCR, 10% VGPR, and 50% PR, followed by 91.6% with negative MRD status at 10^−5^ sensitivity. 86% of patients developed CRS with a median onset of two days after the first infusion and a median duration of four days. All cases were grade 1 or 2 with no grade > 3 events and were resolved by the treatment of tocilizumab or corticosteroid. In addition, one patient experienced grade 2 neurotoxicity, which completely resolved within 24 h by the treatment of dexamethasone [148] (Table 5).

CRB-402, bb21217, a phase 1 dose escalation trial of bb21217 in 46 RRMM patients was conducted, and BCMA+ CAR-T cells across a dose range of 150–450 × 10^6^/kg were given [149]. The updated results included a 55% ORR with 18% CR, and 30% VGPR. DOR was 11.9 months, which was positively correlated with CD127 expression related to memory T cell formation. CRS with grade 1 or 2 occurred in 67% of patients with a median onset of three days. In contrast, CRS with a grade > 3 occurred only in 4% of patients. Twenty-two percent of patients experienced neurotoxicity, including 6% of grade > 3 events with a median time to onset of seven days [149] (Table 5).

P-MCMA-101 was a second-generation CAR construct manufactured using a non-viral piggyBac gene whose binding domain is not scFv but a small fully human fibronectin domain, known as Centyrin. Phase 1/2 clinical trial in 43 patients with RRMM treated at a dose range of 1.75–15 × 10^6^/kg cells showed an ORR of 57%. CRS was seen in 17% of patients, with only one patient being grade > 3. Grade 1–2 neurotoxic events occurred in four patients [150] (Table 5).

In the phase 2 LEGEND-2 study of LCAR-B38M CAR-T cells in 57 patients with RRMM conducted in China, results showed deep and durable responses with an ORR of 88% and a median PFS of 19.9 months [151]. It also revealed manageable safety profiles. Furthermore, the phase 1b/2 CARTITUDE-1 study of Cilita-cel (JNJ68284528) was evaluated [152]. The updated results included 97 RRMM patients who had received >3 prior lines of therapy or were double refractory to PIs or IMiDs and received anti-CD38 mAb received an infusion of Clilta-Cel at a dose of 0.5–1.0 × 10^6^/kg. They showed an ORR of 96.9% with 67.0% sCR, 25.8% VGPR, and 4.1% PR. In addition, 93.0% achieved MRD negativity at 10^-5^, and a median time to attain MRD negativity was one month, while the 12- month PFS and OS rates were 76.7% and 88.5%, respectively. The CRS were reported to be developed in 94.8% of patients, including 4.1% grade > 3 with a mean onset of seven days. Neurotoxicity occurred 20.6% including 10.3% grade > 3. A phase III study has already been initiated [152] (Table 5).

C-CAR088 therapy in 24 patients revealed a favorable safety profile and a promising clinical result at dose levels of 1.0–6.0 × 10/6kg. The ORR was 95.2% with 28.6% CR + sCR, 10% VGPR and 19% PR; 45% of patients who were infused with 3.0 × 10^6^/kg cells achieved aCR. 95% of patients experienced CRS, which were almost grade 1 or 2, with a median onset of 6.5 days and a median duration of five days [153] (Table 5).

The phase 1/2 study of Orva-cel; JCARH125 at a dose of 50–600 × 10^6/^kg in 52 RRMM patients showed an ORR of 92%, including 36% CR [154] (Table 5).

However, there are several limitations in CAR-T cell therapy in hematological malignancies. In brief, the manufacturing of autologous CAR-T cells limits the number of patients who attain benefits from this therapy [112,113,114]. It is sometimes difficult to correct sufficient numbers of autologous T cells through leukapheresis because the majority of patients show leukopenia in their peripheral blood due to continuous prior chemotherapies. In addition, in several hematological malignancies, it is difficult to generate CAR-T cells in bulk from patients to kill targeted tumor cells because of T cell exhaustion by prior treatments. Disease progression during CAR-T cell manufacturing process, antigen evasion because of the diminished tumor-specific antigen expression on tumor cells, the existence of antigen in the circulation, inhibiting CAR-T cells, and high costs of CAR-T cell manufacturing also become serious issues for patients [112,113,114]. Furthermore, in relapsed or refractory disease, tumor cells may be harvested together with leukocytes during leukapheresis. As a result, tumor cells might be transduced with CAR construct and administrated into patients. Thus, administrated CAR-cells expand in vivo, and CARs expressed in tumor cells might contribute to antigen escape by the down-regulation of tumor-specific antigen expression [112,113,114].

Especially, despite the initial high response rates, loss or down-regulation of targeted-antigen expression in tumor cells is observed in a subset of patients treated with CAR-T cells. In 11–25% of patients with CD19 positive B-ALL, treated with CD19 CAR-T cells were reported to have been relapsed as CD19 negative disease [155]. The existence of CD19 splice variants was reported to lead to antigen escape by CD19 CAR-T cells in patients with B-ALL. Especially, two mechanisms, CD19 splice variants, which specifically lacks an exon containing the extracellular epitope of CD19, and variants that lack the transmembrane domain of CD19, leading to decreased surface CD19 expression were detected [156]. Moreover, loss of CD81, a chaperon protein of CD19, was reported to be the cause of CD19 loss after the treatment of blinatumomab in B-ALL [157].

To improve the efficacy of CAR-T cells, the challenges of developing the next generation of CARs include getting multi-specific CAR-T cells to respond to the lower levels of targeted-antigen on tumor cells as well as to reveal more efficient induction of anti-tumor immune response. There were several methods to generate bi-specific CAR-T cells with multi-specificity. Tandem CAR-T cells were engineered using a single vector that encodes a bivalent CAR molecule which recognizes two different targeted-antigens in tumor cells. The bicistronic CAR-T cells were generated with a single vector that encodes two independent CAR molecules, each recognizing different targets [158].

In CAR-T cell therapy of MM, bi-specific CAR-T cells with a single CAR molecule attached to two or more different binding domains were engineered. Targeting CD19 can trigger the elimination of tumor cells by CAR-T cells, so targeting both BCMA and CD19 antigens might improve efficacy and reduce relapse [159]. A multicenter study of human dual BCMA/CD19 targeted CAR-T cells, GC012F for 16 heavily pretreated RRMM patients with a median of five prior lines of therapy revealed an early and high response with an ORR of 93.8%, including 56.3%, sCR + CR, and 37.5% VGPR. All of the patients who received the therapy at the highest dose level achieved 100% MRD negativity at 10^−4^ or 10^−6^ levels at three months [159]. The expansion and persistence of CAR-T cells were attained in all patients, dependent on infused cell doses, and were also detectable beyond 28 weeks. GC012F showed an acceptable safety profile, and observed AEs were manageable; 87.5% of patients who developed grade 1–2 CRS and 12.5% who developed grade 3, with a median onset of six days, were successfully treated with tocilizumab, dexamethasone, or vasopressors with a median duration of four days. Neurotoxicity such as ICANS did not occur at any grade [160]. Altogether, GC012F showed promising activity with deeper, faster, and more durable responses in RRMM patients (Table 5).

Furthermore, multi-target CAR-T cell therapies were created by mixing multiple CAR-T cells with a single CAR to target a different tumor-specific antigen such as BCMA + CD19, BCMA + GPRC5D, BCMA + TACO, and BCMA + SLAMF7 [159,161]. These CAR-T cells were engineered independently and co-administrated together or sequentially. Dual infusion of humanized anti-CD19 CAR-T cells (1.0 × 10^6^/kg) and anti-BCMA CAR-T cells (1.0 × 10^6^/kg) was performed in 21 patients with RRMM; 95% of patients achieved an ORR including 43% sCR, 14% CR, 24% VGPR, and 14% PR, and 81% of patients also showed MRD negativity [161]. AEs include CRS, which occurred in 90% of patients, including 86% patients with grade 1 or 2 and 5% with grade 3, hematological toxicities such as leukopenia, anemia, and thrombocytopenia or immunological toxicities such as cryoglobulinemia and B cell aphasia were experienced in 95% of patients with 86% patients experiencing > grade 3 [161] (Table 5).

As an alternative therapeutic approach, CAR-T cells, engineered from allogenic donor T cells, were used. Indeed, leukocytes were collected from an HLA-matched single donor, genetically modified to express anti-CD19 CAR, then allogenic anti-CD19 CAR-T cells were generated. The administration of anti-CD19 CAR-T cells into patients with persisting B cell malignancies after allogenic-SCT showed rapid regression of tumors without any evidence of graft-versus-host disease (GVHD), even if patients were refractory to donor lymphocyte infusions (DLIs) [162]. These results imply that allogenic CAR-T cells, genetically modified to express a tumor-specific antigen as well as CRISPER/Cas9 techniques used to generate allogenic universal CAR-T cells by replacing endogenous TCRs locus with CAR in T cells, are promising therapeutic approach for the cure for MM [163]. Moreover, both higher dose levels and increased persistence of CAR-T cells in the blood are required to show their efficacy, therefore infused dose-escalation, repeated infusion, and alteration or the addition of co-stimulatory molecules to CAR-T cells have to be considered. Actually, to increase CAR-T cell persistence, CAR-T cells manufactured by less differentiated T cells such as naive T cells and central memory T cells had a greater proliferative capacity and had more potent cytotoxicity than traditional CAR-T cells [164]. In addition, armored CAR-T cells include a CAR with co-expression of both CD28 and 4-1BB in the costimulatory domain, IL-18 secreting CARs to induce enhanced proliferation and prolonged persistence, PD-1 blocking scFv secreting CARs, IL-18 secreting CARs, and CARs including CD40 ligand to stimulate anti-tumor response [165].

Moreover, the host immune system recognizes and eliminates donor T cells. So, the engraftment of allogenic donor T cells causes the host to yield GVHD. Thus, the expression of CARs in alternative allogenic donor cell types, such as CAR-NK cells that avoid the development of GVHD more efficiently. On the other hand, it was also reported that the existence of CAR-NK cells in blood circulation reduced their persistence. Therefore, further investigation is needed to validate these results [166].

### 3.1. CAR-NK Cells

More than 85% of NK cells present as CD56^dim^ CD16^+^cells and are functionally similar to CD8 positive cytotoxic T cells in their mechanisms of action during the killing of targeted cells. Activated NK cells chiefly revealed ADCC to induce their cytotoxicity and also secreted a variety of inflammatory cytokines or chemokines to activate other immune cells, which are engineered to express T cell receptor (TCR) or CARs to recognize and kill targeted tumor cells. CAR-NK cells offer several significant benefits, compared with CAR-T cells, including the convenience of providing off-the-shelf products, safety, to reduce the risk of on- and off-tumor adverse effects, and induction of cytotoxicity against tumor cells via multiple mechanisms of action [167,168].

CAR-T cell therapy is a personalized therapy using patient-specific CAR-T cells, while CAR-NK cells decreased the risk for alloreactivity, resulting in the reduction of the development of GVHD, which allow CAR-NK cell products to be generated from multiple allogenic cell sources, including NK cell lines; NK92 or NK92MI, PBMCs, umbilical cord blood cells; UBCs, induced pluripotent stem cells; iPSCs and CD34+ HPCs [167,168]. NK92-derived CAR-NK cells have been tested in a variety of clinical trials, which have shown benefits in requiring less manufacturing time and having lower costs. PBMC-derived CAR-NK cells with the expression of CD56^dim^ and CD16^+^, characteristics of a mature phenotype, yielded increased cytotoxicity. Umblical cord blood (UCB)-derived CAR-NK cells, showing an immature phenotype with a reduced expression of CD16, perforin, and granzyme B, but an increased expression of an inhibitory molecule, NKG2A, reduced the cytotoxicity against tumor cells [167,168]. In addition, the low number of UCB-derived NK cells collected from a UCB bank limited the advantage in their clinical use. In the manufactures of iPSCs-derived CAR-NK cells, iPSCs could be more efficiently engineered to express a CAR, compared with mature NK cells. First, CAR-engineered iPSCs are cultured with various cytokines such as SCF, VEGF, and BMP4 and HPCs are generated. Afterward, these HPCs are stimulated with IL-3, IL-7, IL-15, SCF, and FLT3L to differentiate into homogeneous CAR-NK cells with an immature phenotype, which showed potent cytotoxicity against tumor cells and could be used clinically [167,168].

During the manufacturing process, an efficient gene transfer system is lacking in NK cells compared with T cells. The efficiency of lentiviral transduction is low in NK cells, which needs repeated transductions [169]. On the other hand, high efficiency of gene transfer has been attained in NK cells transduced with a single retrovirus [170]. Therefore, retroviral gene transduction is extensively used to generate CAR-NK cells for clinical use. However, retroviral-based transduction does not represent a safety concern due to the risk of the development of genomic instability, including mutagenesis in NK cells [171,172].

To attain efficient expansion and persistence of CAR-NK cells, the second-generation anti-CD19 CARs containing scFv, CD8, 4-1BB, and CD3Zeta were engineered using primary NK cells in B-ALL [173]. Moreover, NK cells with 2B4 in NK cell-specific co-stimulatory domain revealed enhanced cytotoxicity compared with CAR-NK cells containing 4-1B or CD28 alone [174]. Although CAR-NK cell proliferative capacity induced by several cytokines was limited in vitro, compared with CAR-T cells [174], memory-like NK cells induced by the stimulation with IL-12, IL-15, and IL-18 increased their expansion [175]. Co-culture of NK cells with feeder cells including autologous PBMCs or chronic myeloid leukemia cell lines; K562 also induced a rapid expansion and prolonged persistence of NK cells for 8-15 weeks [176,177].

CAR-NK cells have a safety profile with reduced on-tumor and off-tumor toxicities towards normal cells. Because the life span of CAR-T cells is relatively short and cytokines secreted by CAR-NK cells also differ from those by CAR-T cells, severe CRS and ICANS are less likely to occur by allogenic CAR-NK cell infusion [129,130,131,132,167,168].

CAR-NK cells can efficiently eradicate tumor cells via both CAR-mediated direct cytotoxicity and CAR-independent NK cell-mediated ADCC. Therefore, even if escape of CAR-targeted antigen expression in tumor cells occurs, CAR-NK cells can attack tumor cells in a CAR-independent manner. Moreover, NK cells expressing a CAR targeting down-regulated tumor-specific antigen on tumor cells can promote NK cells to migrate to tumor cells, leading to NK cell-mediated ADCC, although these cells do not induce direct killing of tumor cells [167,168].

Armored CAR-NK cells were generated using CRISPED/Cas9 gene modification techniques to co-express molecules with CARs on NK cells to secrete cytokines or chemokines to enhance NK cell proliferation or migration and increase maximal cytotoxicity with minimal adverse effects towards normal cells. Moreover, lymphodepleting chemotherapy conducted before the infusion of CAR-NK cells has the potential to modify the tumor microenvironment by depleting immunosuppressive cells, including MDSCs, Tregs, and B regulatory cells (Bregs), resulting in reinforcing anti-immune response and NK cell proliferation [178].

Thus, although the potential of allogenic CAR-NK cells is promising, it has shown only limited progress in clinical settings. The result of a clinical trial with CD19-targeted CAR-NK cell therapy by UCB-derived CAR-NK cells for 11 patients with CD19 positive B cell lymphoma and CLL has recently demonstrated promising clinical efficacy, feasibility, and safety profiles. It was reported that seven of 11 patients achieved CR without serious adverse effects [174].

In MM, Anti-CD138 targeting CAR-NK cells derived from NK-92MI revealed a cytotoxic activity [179]. Both CS- and CD138-targeting dual CAR-NK cells from NK-92 suppressed MM cell growth and survival using xenograft models [179,180]. Activated NK cells express T cell immune checkpoint molecules such as PD-1, CTCL-4, TIM-3, and LAG3, which inhibit the NK cell immune response. In the future, blocking antibodies or checkpoint inhibitors might enhance NK cell activity, and the combination therapy of CAR-NK cells with checkpoint inhibitors, blocking antibodies, or gene-editing system to inhibit checkpoint molecules would be promising therapeutic approaches [181,182,183].

### 3.2. Dendritic Cells (DCs)

Dendritic cells (DCs) are antigen-presenting cells that present antigens on the cell surface to naïve T cells and modify the anti-tumor immune response. DCs in patients with MM are dysfunctional, so it is ideal that DCs are generated ex vivo. These DCs work well and are capable of causing CTLs to enact an anti-immune response [184,185]. A combination of DC vaccine plus lenalidomide synergistically enhanced anti-tumor immune response in vivo [186]. Therefore, the combination therapy with DCs plus novel agents including lenalidomide or PD-1 inhibiter may modulate anti-tumor immune deficiency and promote cytotoxicity against MM cells, compared with a DC vaccine alone. Large scales of studies are necessary to validate these results [184,185].

#### 3.2.1. Idiotype (Id) Protein-Pulsed DCs

Idiotype (Id) is a tumor-specific antigen secreted by MM cells [187]. Although vaccination of Id-pulsed DCs activated Id-specific CTLs, which showed an immunological response. However, these DCs tested in clinical trials had disappointing results because the Id-protein was too weakly expressed in MM cells to activate CTLs, and Id-pulsed DCs also targeted only a single tumor antigen [188].

#### 3.2.2. Whole MM Cell-Derived Antigen-Loaded DCs

DCs pulsed with whole MM cells derived from patients with MM could present multiple epitopes to MHC on DCs, leading to the induce polyclonal T cell immune responses [185]. Thus, DCs pulsed with an adequate concentration of MM cell lysates revealed potent cytotoxicity, while a high concentration of tumor lysates inhibited DC function. Thereby, CD138 positive MM cell lysate-pulsed DCs could induce a higher CTL response than a whole MM cell lysate-pulsed DCs [189]. Furthermore, vaccination with DC plus MM cell fusion in patients with RRMM induced both a CD4+ T cell and cytotoxic CTL response, resulting in a stable disease condition [184]. DCs loaded with dying MM cells (VAX-DC/MM) also enhanced the capacity of migration in DCs and succeeded in the generation of MM-specific CTLs [190]. Clinical trials using VAX-DC/MM demonstrated a clinical benefit rate in 66.7% of patients, and 11.1% of patients had a minor response (MR), and 55.6% achieved stable disease (SD) [191].

#### 3.2.3. MM Cell-Specific Antigen-Loaded DCs

A variety of MM-associated antigens were identified in MM. DCs pulsed with MAGE, cancer-testis antigen, sDickkof-1, CD138, CS1, XBP1, survivin, and BCMA induce antigen-specific CTLs targeting MM cells in vitro, and several vaccines have been tested in clinical trials [192,193,194,195,196,197]. CS1-pulsed DCs caused an increase in effector memory T cells and activated CTLs [196]. Antigen mRNA-laded DCs, pulsed with MAGE3, survivin and BCMA promoted tumor-specific CTLs [184,185,193,197].

## 4. Immune Check Point Inhibitors in MM

Targeting immune checkpoints with Programmed cell death protein 1 PD-1/ programmed cell death ligand 1 (PD-L1) pathway inhibitors or blockade of CTLA-4 has proven to be an effective therapeutic approach for various cancers. The interaction between PD-1 on immune cells and its ligand, PD-L1 on tumor cells, deliver an inhibitory signal to immune cells, leading to T cell anergy, thereby tumor cells evade the immune system.

Indeed, the blockade of the PD-1/PD-L1 pathway is a highly effective therapeutic approach for the majority of patients with classical HL, characterized by an overexpression of PD-L1 in tumor cells due to the alteration in chromosome 9p24.1 [197,198]. Nivolumab (Opdivo^R^), a fully human IgG_4_ anti-PD-1 mAb which interrupts PD-1 activation, leading to an enhanced immune response against tumor cells, was approved in 2016 for the treatment of relapsed or refractory HL [199]. Therefore, the blockade of the PD-1/PD-L1 pathway by regulating immune suppression may also reveal therapeutic efficacy in MM [51,197,198].

PD-L1 is highly expressed in MM cells and is associated with clonal evolution from MGUS to MM or drug resistance in RRMM. In a xenograft model of MM, high expression levels of PD-1 in both CD4+ and CD8+ T cells were observed, compared with control mice; thereby, PD-1 blockade prolonged the survival of MM-bearing mice by depleting CD4+ and CD8+ T cells [51,199]. These results indicate that the PD-1/PD-L1 pathway contributes to the immune evasion and that the blockade is an effective therapeutic approach in MM. By contrast, currently, the outcome of checkpoint blockade with PD-1/PD-L1 inhibitors alone is unsatisfactory in MM, compared with HL or solid tumors due to the reduced immune dysfunction in MM [200]. Therefore, only limited data exist from clinical trials using anti-PD1/PDL1 mAbs in MM. A phase Ib clinical trial of nivolumab monotherapy in 27 RRMM patients showed no objective responses [201]. A phase Ib trial of pembrolizumab, humanized IgGκanti-PD-1 mAb monotherapy, revealed that 17 patients achieved the best ORR with a median duration of response of 3.7 months [202]. On the other hand, lenalidomide downregulates PD-1 expression in immune cells and enhances effector-mediated cytotoxicity against MM. Lenalidomide plus PD-1/PD-L1 inhibitors in combination synergistically increased cytotoxic effects against MM cells via interferon γ secretion by effector cells as well as promoted direct apoptotic MM cell death [203]. A phase I dose-escalation study of pembrolizumab with lenalidomide plus dexamethasone (Rd) in 40 RRMM patients revealed an ORR of 50% with an ORR of 38% in lenalidomide-refractory patients [204]. Phase II clinical trials in 48 RRMM with pomalidomide plus dexamethsone showed an ORR of 60%, including 8% CR and 19% VGPR, with a median duration of response of 14.7 months [205]. These results lead to the development of phase III studies of pembrolizumab with Rd (KEYNOTE-185) or pomalidomide (Pom) plus dexamethasone (Pd) (KEYNOTE-183) [206,207]. However, trials were discontinued in 2017 due to the increased rate of death and no significant difference in an ORR between pembrolizumab monotherapy and its combinations.

## 5. Conclusions

Immune dysregulation plays a crucial role in myeloma pathogenesis and has an influence on the response to immune therapies. Therefore, the development of treatment strategies targeting immune defects is important to augment and restore anti-MM T cell responses as well as generate deep and durable responses in MM.

The rapid evolution of cellular immunotherapies with mAbs, ADCs, BsAbs, and autologous or allogenic CAR-T or NK cells and their combinations has already started to reshape the treatment paradigm of RRMM.

## Figures and Tables

**Table 1:** Mechanisms of action in anti-CD38 monoclonal antibodies.

mAb	Daratumumab	Isatuximab
Isotype	IgG1λ	IgG1κ
Origin	human	chimeric
Administration route	IV, SC	IV
ADDC	+	++
CDC	++	+
ADCP	+	+
Programmed direct cell death	−	++
Cross linking	+	−
Ectoenzymic activity	+	++

mAb, monoclonal antibody; IV, intraveneously; SC, subcutaneously; ADCC, antibody dependent cytotoxicity; CDC, complement dependent cytotoxicity; ADCP, antibody dependent cellular phagocytosis.

**Table 2:** Bispecific antibody (BsAb) in MM.

Agent	Target	Phase	Response	Adverse Effects-Grade 1, 2 (>3)
AMG420	BCMA × CD3	I	ORR * 70%/31%, DOR 9	CRS 38(2)%, Infections 33(24)%
CC-93269	BCMA × CD3	I	ORR ″ 89%/36%	CRS 77(3)%, Infections (26)%, 0%
PF-06863135	BCMA × CD3	I	ORR 75%	CRS 61(0)%, IRS 33(0)%
REGN5458	BCMA × CD3	I/II	ORR ^#^ 62%/35.6%, DOR 6.0 m	CRS 39(0)%, Infection 47(18)%, NTX 12(0)%
AMG701	BCMA × CD3	I/II	ORR 36%, DOR 3.8 m	CRS 61(7)%, Infections (17)%, NTX 8(0)%
TNB-383B	BCMA × CD3	I	ORR ^$^ 80%, DOR 4.5 m	CRS 45(0)%, Infections 21%
Teclistamab/JNJ-64007957	BCMA × CD3	I	ORR 69%, DOR NR	CRS 55(0)%, Infections 52(15)%, NTX 5(2)%
GBR1342	CD38 × CD3	I	On going until 2021	-
AMG424	CD38 × CD3	I	On going until 2022	-
Talquetamab/JNJ-64407564	GPRC5D × CD3	I	ORR ^&^ 69%/63%, DOR NR	CRS 68(10)%, Infections 38(8)%, Skin 45%, NTX 6(2)%
Cevostamab/BFCR4350A	FcRH5 × CD3	I	ORR ^|^ 53%, DOR 6 m	CRS 76(2)%, NTX > 2%

BCMA, B-cell maturation antigen; BsAb, Bispecific antibody; GPRC5D, G-Protein-coupled Receptor Class 5 Member D; FCRH5, Fc Receptor Homolog; ORR, overall response rate; DOR, Duration of response; CRS, cytokine release syndrome; NTX, neurotoxicity excluding CRS, * AMG420 at the dose of 400 μg/kg/across all doses, ″ CC-93269 at doses >6 mg/3–6 mg, ^#^ REGN5458 at doses #60 mg/##across all doses, ^$^ TNB383B at doses 40 mg, ^&^ Talquetamab at doses 450 μg/kg SC(RP2D)/across all doses, ^|^ Cevostamab at doses 3.6 mg/20 mg.

**Table 3:** Antibody drug conjugate (ADC) in MM.

Agents	Target	Toxin	Phase	Response
Belanatmab mafadotin/GSK2857916	BCMA	MMAF	I(DREAMM-1)	ORR 60%, PFS 12 m, DOR 14.3 m
	BCMA	MMAF	II(DREAMM-2)	ORR 34%/31%, PFS 4.9 m/2.9 m
	BCMA	MMAF	I/II(DREAMM-4)	ORR 67%, PFS NR
	BCMA	MMMAF	I/II(DREAMM-6)	ORR 78%, PFS NR
MEDI2228	BCMA	PBD	I	ORR 61%, PFS NR
AMG224	BCMA	Mertansine	I	ORR 23%
CC99712	BCMA	MMAE	I	Not results yet
TAK-169	CD38	Shiga like toxin	I	Not results yet
TAK-573	CD38	IFNα2b	I	ORR 7%
Indatuximab ravtansine/BT062	CD138	Ravtansine	I/II	ORR 78% (with Rd)
Lorvotuzumab mertanisine/IMGN901	CD56	Mertansine	I/IIa, I/IIb	ORR 5.7%, SD 42.9%, DOR 15.8 m
Milatuzumab doxorubicin/STRO-001	CD74	Doxorubicin	I	No ORR, SD 26% (1/14 Pts)
FOR46	CD46	MMAE	I	Not results yet

ADC, antibody drug conjugate; BCMA, B-cell maturation antigen; BAFF, B-cell activating factor; MMAF, Monomethyl auristatin F; PBD, Pyrrolobenzodiazepine; MMAE, Monomethyl auristatin E; ORR, overall response rate; PFS, Progression free survival; DOR, duration of response; Rd, lenalidomide+dexamethasone; SD, stable disease.

**Table 4:** Novel targets in chimeric antigen receptor (CAR)-T cell therapy in MM.

Target	CAR Construct	Costimulatory Molecule	MM Cells	Normal Cells	Clinical Trials
CD19	murine scFv	4-1BB	+/− or −	B cell	Done; NR or MR
CD38	murine scFv	CD28	++	B, T, NK, plasma cell, osteoclast	Not yet reported
CD44v6	human	CD28	+ or −	T cell, monocyte	Not ret reported
CD70	human	CD28, 4-1BB	+ or −	B, T cell	Done
CD138	murine scFv	CD28	++	Plasma cell	Not yet reported
Igκ	murine scFv	CD28	+ or −	B cell	Done; SD
SLAMF7/CS1		CD28, 4-1BB	++	B, T, NK, plasma cell, monocyte	Not yet reported
BCMA/	murine scFv	CD28, 4-1BB	++	B cell, plasma cell	Reported (Table 5)
CD269	human scFv				
Integrinβ7	human scFv	CD28	+	B, T cell	Not yet reported

CAR, chimeric antigen receptor, MM, multiple myeloma; scFv, single—chain variable fragments; Igκ, immunoglobulin light chain; SLAMF7, signaling lymphocyte-activating molecule F7; BCMA, B-cell maturation antigen; NR, no response; MR, minimal response; SD, stable disease.

**Table 5:** BCMA-targeted CAR-T cell therapy in relapsed or refractory MM.

Trial	Target	Activation Domain	Binding Domain	Phase	Responses	Outcome	CRS Gr > 3	NTX Gr > 3
CRB401/ KarMMa	Ide-cel/ bb2121	4-1BB	Murine scFv	1/2	ORR 85%, CR 9%, sCR 36%	PFS 11.8 m	76% 6%	42% 3%
CRB-401	Ide-cel/bb2121	4-1BB	Murine scFv	1	ORR 76%, DOR 10.3 m	PFS 8.8 m OS 34.2 m	76% 6%	44% 3%
CT053	CT053	4-1BB	Human scFv	1	ORR 87.5%,CR 12.5%, sCR 66.7%	PFS 18.8 m DOR 21.8 m	62.5% 0%	4% 4%
LUMMICAR-2	CT053	4-1BB	Human scFv	1b/2	ORR 100%, sCR 20% CR 20%	-	86% 0%	7% 7%
PRIME	P-BCMA-101/Poseida	4-1BB	Centyrin human	1/2	ORR 57%	PFS NR	17% 2%	9% -
C-CAR088	C-CAR088	4-1BB	Human scFv	1/2	ORR 95.2%, sCR + CR 28.6%, VGPR 10%	DOR NR	95% 4%	4% -
CRB-402	Ide-cel bb21217	4-1BB PI3Ki	Murine scFv	1	ORR 55%, CR 18%, VGPR 30%	DOR 11.9 m	67% 4%	22% 6%
LEGEND-2	CAR-B38M	4-1BB	Human scFv	1	ORR 88%	PFS 19.9 m	- -	- -
CARTITUD	Cilta-cel/JNJ-68284528	4-1BB	IIama-dual VHH	1b/2	ORR 96.9%, sCR 67.0%, VGPR 25.8%, MRDneg at 10-5 93%	PFS NR, DOR NR	94% 4%	20% 10%
EVOLVE	Orva-cel CARH125	4-1BB	Human scFv	1/2	ORR 92%, CR 36%	PFS NR	80% 2%	25% 7%
ChiCTR	CD19CAR/BCMA CAR	4-1BB	Human scFv	1	ORR 95%, CR 43% MRD neg 81%	-	90% 4%	- -
GC012F	BCMA-CD19dual FasT CAR-T	4-1BB	Human scFv	1	ORR 93.8%, sCR + CR 56.3%, VGPR 23.8% MRDneg at 10-4s	DOR NR	87.5% 12.5%	- -

CAR, chimeric antigen receptor, MM, multiple myeloma; scFv, single chain variable fragments; BCMA, B-cell maturation antigen; CRS, cytokine release syndrome; NTX, neurotoxicity; ORR, overall response rate; CR, complete remission; sCR, stringent complete remission; MRD, minimal residual disease.
